# Effects of Lidocaine Oropharyngeal Spray Applied Before Endotracheal Intubation on QT Dispersion in Patients Undergoing Coronary Artery Bypass Grafting: A Prospective Randomized Controlled Study

**DOI:** 10.21470/1678-9741-2019-0112

**Published:** 2020

**Authors:** Murat Bilgi, Yusuf Velioglu, Hamit Yoldas, Mehmet Cosgun, Ahmet Yuksel, Ibrahim Karagoz, Isa Yildiz, Abdulhamit Es, Duygu Caliskan, Kemalettin Erdem, Abdullah Demirhan

**Affiliations:** 1 Abant Izzet Baysal University Medical School, Bolu, Turkey.; 2 Abant Izzet Baysal University Faculty of Economics and Administrative Sciences, Bolu, Turkey.

**Keywords:** Topical Lidocaine, QT Dispersion, Coronary Artery Bypass Grafts, CABG, Hemodynamic Response

## Abstract

**Objective:**

To investigate the effects of lidocaine oropharyngeal spray applied before endotracheal intubation on hemodynamic responses and electrocardiographic parameters in patients undergoing coronary artery bypass grafting.

**Methods:**

A total of 60 patients who underwent coronary artery bypass grafting surgery were included in this prospective randomized controlled study. Patients were randomly divided into two groups, the topical lidocaine group (administration of 10% lidocaine oropharyngeal spray, five minutes before laryngoscopy and endotracheal intubation) and the control group. Both groups were compared with each other in terms of main hemodynamic parameters including mean arterial pressure and heart rate, as well as *P* and QT wave dispersion durations, before and after endotracheal intubation.

**Results:**

The groups were similar in terms of age, gender, and other demographics and basic clinical characteristics. There was a statistically significant difference between the groups in terms of QT dispersion durations after laryngoscopy and endotracheal intubation. The increase in QT dispersion duration was not statistically significant in the topical lidocaine group, whereas the increase in QT dispersion duration was statistically significant in the control group. When the groups were compared in terms of *P* wave dispersion durations, there were significant decreases in both groups, but there was no significant difference between the groups.

**Conclusion:**

Our study revealed that the topical lidocaine administration before endotracheal intubation prevented increase of QT dispersion duration in patients undergoing coronary artery bypass grafting.

**Trial Registration:**

NCT03304431

**Table:** 

Abbreviations, acronyms & symbols				
ASA	= American Society of Anesthesiologists		IV	= Intravenous
BMI	= Body mass index		MAP	= Mean arterial pressure
CABG	= Coronary artery bypass grafting		Pd	= *P* wave durations
CONSORT	= Consolidated Standards of Reporting Trials		Pwd	= *P* wave dispersion
COPD	= Chronic pulmonary obstructive disease		QTc	= Corrected QT
DM	= Diabetes mellitus		QTd	= QT dispersion
ECG	= Electrocardiography		SpO_2_	= Peripheral oxygen saturation
HR	= Heart rate		SPSS	= Statistical Package for the Social Sciences
HT	= Hypertension			

## INTRODUCTION

QT duration on electrogram reflects the total duration lasting for depolarization and repolarization of the ventricles, while *P* wave duration (Pd) reflects the atrial contractions. QT duration is the time interval between the beginning of Q wave and the end of T wave, while Pd was defined as the time interval between the beginning of *P* wave and the returning of the wave to the isoelectric line. QT dispersion (QTd) was obtained by measurement of the difference between the longest and the shortest QT duration in all derivations, while *P* wave dispersion (Pwd) was recorded as the difference between the longest and the shortest Pd in all derivations. Prolongation of these durations may cause cardiac adverse events^[1,2]^. The reasons of prolonged QT duration may include congenital cardiac disorders, electrolyte disturbances, advanged age, female gender, drugs, sympathetic activity, and increased catecholamine concentrations^[3-5]^. The sympathetic activity and increased catecholamine concentrations that occur in response to endotracheal intubation may cause life-threatening cardiac events, especially in patients undergoing major cardiac surgeries, such as coronary artery bypass grafting (CABG)^[6,7]^. Increased sympathoadrenal activity as a result of laryngoscopy and endotracheal intubation is known to prolong QT duration, QTd, and Pwd on electrocardiography (ECG). Previous studies have shown that these alterations on ECG cause life-threatening ventricular and atrial arrhythmias^[8-11]^. Some pharmacological agents, including opioid analgesics (such as fentanyl and alfentanil), beta blockers, and intravenous (IV) lidocaine, are administered before endotracheal intubation in order to prevent hypertension and tachycardia that may develop following endotracheal intubation^[8,12,13]^. IV lidocaine administration before endotracheal intubation may suppress the hemodynamic response secondary to intubation^[14,15]^. Topical lidocaine (10% lidocaine spray) applied to the oropharyngeal mucosa and supraglottic region may suppress the cough reflex and provide awake fiberoptic intubation^[16]^. As it is known, the most primary cause of cardiovascular response during laryngoscopy and endotracheal intubation is the compression on the supraglottic region by the laryngoscope blade^[17]^. Stimuli arising from this region as a result of the supraglottic compression create sympathetic response by transmitting via vagal and glossopharyngeal nerves. We presume that the effect of this compression on the supraglottic region can be suppressed with topical lidocaine administration, hereby the hemodynamic stress response can decrease, and this situation can positively affect QTd on ECG.

The objective of this study was to investigate the effects of 10% topical lidocaine oropharyngeal spray applied before endotracheal intubation on hemodynamic parameters, including mean arterial pressure (MAP) and heart rate (HR), and electrocardiographic parameters, including QT, QTd, and Pwd durations, in patients undergoing CABG surgery.

## METHODS

### Study Design and Patients

This prospective randomized controlled study was approved by the Abant Izzet Baysal University local ethics committee (approval date: May 26, 2017; decision no: 2017/65). All patients included in the study were informed about the anesthetic method that would be used and the objective of the study, and their written informed consents were obtained. The inclusion criteria were being at American Society of Anesthesiologists (ASA) level III risk, being scheduled for CABG operation, and being aged between 50-75 years. The patients included in this study were selected consecutively among those undergoing elective, first-time, isolated CABG. Patients with cardiomyopathy, cardiac valve disease, arrhythmias, preoperative electrolyte disturbances, medical treatment which may prolong QT duration, those with chronic hepatic or renal dysfunction, patients with a history of allergy to lidocaine, and those with a difficult airway (*e.g*., patients in whom tracheal intubation could not be performed in a single time and those with a laryngoscopy duration exceeding 30 seconds) were excluded from the study. In addition, patients with artifact and arrhythmias during ECG analysis and those with a poor-quality ECG were also excluded from the study.

The patients were randomly divided into two groups via a certified true randomizer number generator (www.random.org, Android Play Store). The groups were named as Group L (which was applied 10% topical lidocaine) and Group C (control group). Following the randomization of the patients, those in Group L were anesthetized with 160 mg 10% topical lidocaine (lidocaine pump oropharyngeal spray 10% 50 mL, 10 mg/puffs, max dose 200 mg) before laryngoscopy and endotracheal intubation. During the topical lidocain application, patients were asked to maximally put out their tongues, and hereby topical anesthesia of the supraglottic region was provided by lidocain oropharyngeal spray towards the glottis at the tongue root and around.

After two wide peripheral branula (18 gauge) were inserted into the different arms’ veins of patients transferred to the operation room, premedication was performed with 0.03 mg/kg of IV midazolam (Dormicum 1mg/mL, Roche preparations Inc., Istanbul, Turkey). And 2 L/min of O_2_ were applied to all patients with a nasal cannula. After topical anesthesia, Allen test was performed with a 20 G cannula, and then radial artery at the suitable arm was cannulated to achieve continuous arterial blood pressure monitoring. Baseline preoperative data of hemodynamic parameters including MAP, HR, peripheral oxygen saturation (SpO_2_), and ECG were recorded. ECG was recorded by using a 12-lead ECG device (velocity: 25 mm/sec, amplitude: 10 mm/mV) (Nihon Kohden, Model ECG-1350K, Japan).

The primary endpoint of the study was QTd, while the secondary endpoints were Pwd, MAP, and HR.

### Anesthetic Management

During induction of general anesthesia, 2 µg/kg fentanyl (Talinat 50 mcg/ml VEM Ilac San. ve Tic. A.S. Istanbul, Turkey), 2 mg/kg propofol (Propofol 10 mg/mL, Fresenius Kabi AB Uppsala/Sweden), and 0.6 mg/kg rocuronium bromide (Muscuran 10 mg/mL Kocak Farma Ilac ve Kimya Sanayi A.S. Tekirdag, Turkey) were administered in both groups. After providing sufficient muscle relaxation following the induction, laryngoscopy and endotracheal intubation (with no. 8 endotracheal tube) were performed by an experienced anesthesiologist. Anesthesia maintenance was provided with 2% sevoflurane in 50% air and 50% oxygen. ECG records were obtained in four time periods: before intubation (baseline), after the induction, and at the 1^st^ and 3^rd^ minutes of the intubation. Hemodynamic measurements including MAP, HR, and SpO_2_ were recorded at baseline (T0), 1^st^ minute of anesthetic induction (T1), and 30^th^ second (T2), 1^st^ minute (T3), 2^nd^ minute (T4), 3^rd^ minute (T5), 4^th^ minute (T6), 5^th^ minute (T7), and 10^th^ minute (T8) of the intubation.

### ECG Analysis

ECG records were transferred to digital media for analysis. ECG analysis and hemodynamic data recordings were performed by a researcher who was blinded to the groups. QT duration was defined as the time interval between the beginning of Q wave and the end of T wave, while Pd was defined as the time interval between the beginning of *P* wave and the returning of the wave to the isoelectric line. QTd was obtained by measurement of the difference between the longest and the shortest QT duration in all derivations, while Pwd was recorded as the difference between the longest and the shortest Pd in all derivations. The data (QT duration, QTd, Pd, Pwd) were extracted using a screen ruler, with the assist of https://www.arulerforwindows.com, and recorded as milliseconds (ms).

### Sample Size Calculation

The primary endpoint of our study was the changes in QTd duration after intubation. Sample size estimation was based on the method described by Kaneko et al.^[17]^. In order to detect a 20% change in QTd duration (42.6±5.8 ms control values in the study by Kaneko et al.^[17]^, with an α error of 0.05 and a power of 95%), we found out that the sample size should be at least 27 patients per group. Estimating an approximate 20% dropout rate, we included 30 patients in each group. The sample size estimation was performed using G Power 3 Calculator.

### Statistical Analysis

Data were analyzed using the Statistical Package for the Social Sciences (SPSS) software, version 20.0. Descriptive statistics including age, height, weight, body mass index (BMI), HR, mean MAP, and operational time are expressed as mean ± standard deviation. Normality of the data was tested with Kolmogorov-Smirnov method. In the intergroup comparisons, independent sample *t*-test was used in the analysis of normally distributed variables between the groups, while Mann-Whitney U test was used in the analysis of non-normally distributed variables. In the intragroup comparisons, paired sample *t*-test was used in the analysis of normally distributed variables between the groups, while Wilcoxon’s test was used in the analysis of non-normally distributed variables. The Chi-square and Fisher’s exact tests were used in the analysis of categorical variables. *P*<0.05 values were considered as statistically significant.

## RESULTS

**Fig. 1:**
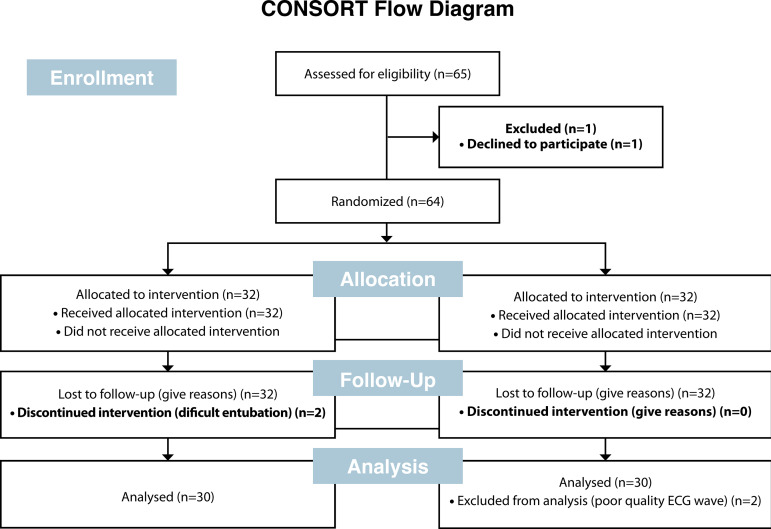
CONSORT flow diagram. CONSORT=Consolidated Standards of Reporting Trials; ECG=electrocardiography

The study initially included 65 patients. One patient rejected to participate in the study. Two patients were excluded from the study due to difficult intubation, and two patients had no sufficient quality of ECG records for the analysis. Hereby, a total of five patients were excluded and 60 patients were included in the study, as shown in the Consolidated Standards of Reporting Trials (CONSORT) flowchart (Figure 1).

There were no statistically significant differences between the groups in terms of demographic data, such as age, height, weight, and BMI (*P*>0.05) (Table 1).

**Table 1:** Baseline characteristics of the groups.

Characteristics	Lidocaine group (n=30)	Control group (n=30)	*P*-value
Age (years)	64.6 (±10.3)	60.3 (±7.5)	0.66
Male, n (%)	22 (73.4%)	27 (90%)	0.09
Female, n (%)	8 (26.6%)	3 (10%)	
Weight (kg)	76 (±11.8)	76.4 (±12.7)	0.89
Height (cm)	166 (±9.0)	169 (±6.1)	0.20
Body mass index (kg/m^2^)	27.3 (±4.3)	26.6 (±3.3)	0.44
DM alone, n (%)	2 (6.6%)	3 (10 %)	0.57
HT alone, n (%)	5 (16.6%)	5 (16.6%)	0.70
DM + HT, n (%)	10 (33.3%)	11 (36.6%)	0.95
Smoking, n (%)	10 (33.3%)	12 (%40)	0.54
COPD, n (%)	3 (10 %)	4 (13.3%)	0.62
Hyperthyroidism	3 (10 %)	4 (13.3%)	0.76

COPD=chronic pulmonary obstructive disease; DM=diabetes mellitus; HT=hypertension →Values are expressed as mean (standard deviation) or n (%).

In the intragroup comparisons of QTd duration, baseline QTd duration was significantly decreased in Group C at the 1^st^ minute after the induction, while this duration was significantly increased at the 1^st^ and 3^rd^ minutes after the intubation (*P*=0.002, *P*=0.000, and *P*=0.016; respectively) (Table 2).

**Table 2:** Electrocardiographic data of the groups.

		T0	T1	T2	T3
QTd (ms)	Group C (n=30)	52.8(±10.1)	48.1 (±8.5)^+^	63.2 (±19.6)^+^	57.4 (±13.5)^+^
	Group L (n=30)	48.6 (±9.2)	46.6 (±8.8)	51.5 (±12.8)*	47.4 (±16.2)*
Pwd (ms)	Group C (n=30)	44.6(±9.1)	36.6. (±9.2)^++^	41.4 (±12.7)	37.7(±10.5)^++^
	Group L (n=30)	46.8 (±10.8)	37.4 (±9.3)^&^	43.4 (±15.1)^&^	36.2 (±12.5)^&^

T0=basal; T1=1^st^ min of induction; T2=1^st^ min of intubation; T3=3^rd^ minute of intubation ms=millisecond; Pwd=*P* wave dispersion; QTd=QT dispersion

* There was a statistically significant difference between Group C and Group L (*P*<0.05)

+ There was a statistically significant difference between Group C QTd basal and the 1^st^ min of induction,1^st^ min of intubation, and 3^rd^ min of intubation (*P*<0.05)

++ There was a statistically significant difference between Group C Pwd duration basal and the 1^st^ min of induction and 3^rd^ min of intubation (*P*<0.05)

& There was a statistically significant difference between Group L basal and the 1^st^ min of induction,1^st^ min of intubation, and 3^rd^ min of intubation (*P*<0.05)

There was no statistically significant difference in baseline QTd duration in Group L compared to the values at the 1^st^ minute after the induction and at the 1^st^ and 3^rd^ minutes after the intubation (*P*>0.05) (Table 2). When both groups were compared in terms of QTd duration, there was a statistically significant difference at the 1^st^ and 3^rd^ minutes of the intubation (*P*<0.05) (Table 2).

**Fig. 2:**
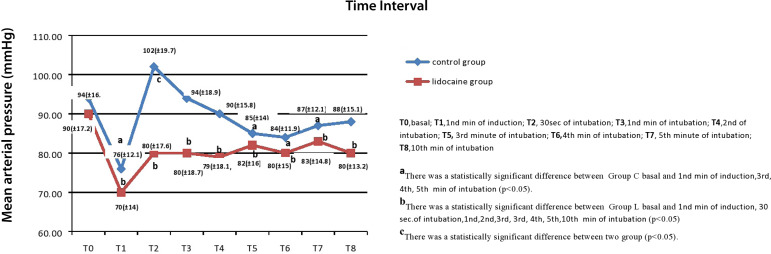
Comparison of mean arterial pressure between control group and lidocaine group.

In the intragroup comparison of changes in MAP, baseline values in Group C were statistically significantly decreased at the 1^st^ minute of the induction, and the 3^rd^, 4^th^, and 5^th^ minutes of the intubation (*P*=0.000, *P*=0.032, *P*=0.015, and *P*=0.030; respectively) (Figure 2). When changes in MAP were compared in Group L, there were statistically significant differences between baseline MAP values and the MAP values at the 30^th^ second and the 1^th^, 2^nd^, 3^rd^, 4^th^, 5^th^, and 10^th^ minutes of the intubation (*P*=0.000, *P*=0.006, *P*=0.003, *P*=0.002, *P*=0.007, *P*=0.007, *P*=0.029, and *P*=0.03; respectively) (Figure 2).

When both groups were compared in terms of changes in MAP values, these values were statistically significantly increased in Group C compared to Group L at the 30^th^ second and the 1^st^ and 2^nd^ minutes of the intubation (*P*=0.000, *P*=0.006, *P*=0.021; respectively) (Figure 2).

**Fig. 3:**
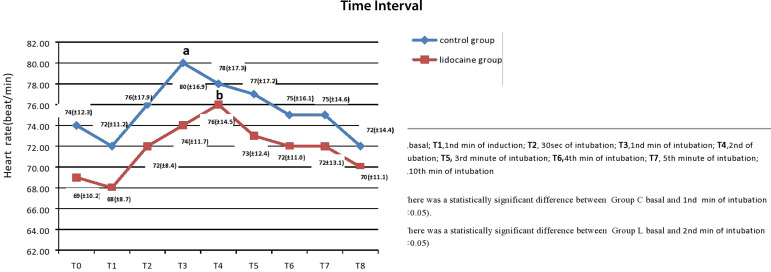
Comparison of heart rate between control group and lidocaine group.

HR was significantly increased in Group C at the 1^st^ minute after the intubation (*P*<0.05), and it was significantly increased in Group L at the 2^nd^ minute after the intubation (*P*<0.05). No significant difference was found between the groups in terms of HR (*P*>0.05) (Figure 3).

In the intragroup comparison of Pwd duration, baseline Pwd duration was significantly decreased at the 1^st^ minute after the induction and 3^rd^ minute after the intubation in Group L (*P*=0.005, *P*=0.015; respectively) (Table 2). Baseline Pwd durations in Group C were statistically significantly decreased at the 1^st^ minute of the induction and the 1^st^ and 3^rd^ minutes of the intubation (*P*=0.003, *P*=0.003, *P*=0.003; respectively) (Table 2). No statistically significant difference was found between both groups in terms of Pwd durations (*P*>0.05) (Table 3).

**Table 3:** Group C and Group L comparisons of Pwd durations.

Pwd duration (ms)	Group C (n=30)	Group L (n=30)	*P*-value
T0	44.6 (±9.1)	46.8 (±10.8)	0.39
T1	36.6 (±9.2)	37.4 (±9.3)	0.74
T2	41.4 (±12.7)	43.4 (±15.1)	0.59
T3	37.7 (±10.5)	36.2 (±12.5)	0.61

T0=basal; T1=1^st^ min of induction; T2=1^st^ min of intubation; T3=3^rd^ minute of intubation No statistically significant difference was found between the groups in terms of Pwd durations (*P*>0.05)

## DISCUSSION

The most important result of this study was that topical lidocaine administration decreased QTd, which was increased after laryngoscopy and endotracheal intubation. Our minor finding was that a stable course was obtained with topical lidocaine administration without sudden increases and decreases in arterial pressure after laryngoscopy and endotracheal intubation.

Patients undergoing CABG operation are usually elderly, with a limited myocardial reserve, and have some comorbidities, such as hypertension and diabetes mellitus^[18-20]^. In our study, the patients’ mean age was 62 years, and male patients were predominant. In patients undergoing CABG with general anesthesia, HR and blood pressure are generally decreased after the induction, while they are increased following laryngoscopy and endotracheal intubation. Those patients whose coronary vessels are occluded and myocardial reserve is limited may not well tolerate conditions that increase the need for oxygen, such as tachycardia and hypertension^[20]^. Laryngoscopy and endotracheal intubation can prepare the ground for severe ventricular and atrial arrhythmias, myocardial ischemia, and infarction^[21,22]^. There are numerous studies in the literature investigating suppression of increased sympathoadrenal response following laryngoscopy and endotracheal intubation^[12,14,22]^. Talwar et al.^[23]^ compared hemodynamic response to intubation following laryngoscopy in patients undergoing elective surgery. The authors administered diltiazem, esmolol, and a mixture of diltiazem and esmolol and consequently reported increased HR following laryngoscopy in the diltiazem and control groups, and decreased HR in the esmolol and mixture groups. In another study evaluating hemodynamic response to laryngoscopy and endotracheal intubation, patients were administered dexmedetomidine, remifentanil, and a mixture of dexmedetomidine and remifentanil. It has been reported that increase in the HR and blood pressure during laryngoscopy was prevented in the mixture group^[24]^. In both studies, it was aimed to blunt hemodynamic response to intubation with İV drug administration before intubation.

In their study, Shribman et al.^[25]^ performed laryngoscopy alone in one group and endotracheal intubation along with laryngoscopy in the other group, and then they compared both groups in terms of the increase in diastolic blood pressure. HR was increased by 24% in the group undergoing laryngoscopy alone, while this increase raised to 36% with addition of endotracheal intubation. As observed in that study, the main cause of sympathoadrenal response after laryngoscopy and endotracheal intubation is the compression on the supraglottic region caused by the laryngoscope blade and the compression on the tracheal mucosa caused by the endotracheal tube^[17,25]^. Topical lidocaine administration may reduce this compression on the supraglottic region, and hereby may prevent sympathoadrenergic discharge^[26]^. In these three studies, it has been emphasized that the mechanical pressure that occurred due to the laryngoscope blade and endotracheal tube can be desensitized with topical anesthesia, and thus sympathetic response can be prevented.

Previous studies have reported that conditions of awake fiberoptic laryngoscopy are provided using topical lidocaine between 100 mg and 200 mg^[2,27,28]^. For the upper respiratory tract, topical lidocaine may be administered at a maximum dose of 200 mg^[28]^. In our study, we administered 160 mg topical lidocaine to provide suitable topical anesthesia. In the present study, significant increase was observed in HR after laryngoscopy and endotracheal intubation in both groups. We believe that the increased HR in the group that we administered topical oropharyngeal lidocaine spray was a result of the compression on the tracheal wall caused by the endotracheal tube. MAP values ranged between 70-83 mmHg during the first 10 minutes in the topical lidocaine group, while these values varied between 76-102 mmHg in the control group. We demonstrated that supraglottic topical lidocaine spray administration prevented fluctuations in MAP after laryngoscopy, leading to a more stable course.

It is important to prevent sympathoadrenal response especially in patients with limited myocardial reserve who will undergo coronary artery surgery. Because, fatal atrial and ventricular arrhythmias secondary to hemodynamic fluctuations occurring following intubation may be observed in these patients^[29]^. Dekker et al.^[30]^ reported increased incidence of coronary heart disease as well as increased risk for myocardial infarction and sudden death in healthy men with prolonged corrected QT (QTc) duration (a QTc duration of 420 ms or higher). Laryngoscopy and endotracheal intubation are known to prolong QTd on ECG^[31]^. Cafiero et al.^[32]^ compared administrations of remifentanil ad bolus during tracheal intubation. The authors reported that remifentanil infusion decreased QT durations. In another study investigating QT durations’ responses to intubation, patients were administered lidocaine, esmolol, and fentanyl before intubation. The authors reported that increases in HR and MAP as well as increases in QT duration following laryngoscopy and endotracheal intubation were prevented in the esmolol group^[32]^. Previous studies reported that İV lidocaine administered before intubation in patients without cardiovascular disease suppressed hemodynamic stress response and prevented prolongation of QTc duration^[5,33,34]^. In the literature screening, we could not find any study examining the effect of topical lidocaine administration on QTd. In our study, we found out that topical lidocaine administration prevented the increase in QTd duration following laryngoscopy and endotracheal intubation.

The main limitation of our study was our relatively small number of patients. A larger study population could increase the statistical power of our study. Another important limitation was that we could not perform an intratracheal analysis to prevent the mucosal compression caused by the tracheal tube, and we could not compare intubation durations between the groups.

## CONCLUSION

We suggest that topical lidocaine administration before laryngoscopy and endotracheal intubation can be useful in patients undergoing CABG since it has hemodynamically beneficial effects and reduces the prolongation of QTd. However, our study should be supported by further studies with larger patient participation.

**Table:** 

Author's roles & responsibilities	
MB	Substantial contributions to the conception or design of the work; or the acquisition, analysis, or interpretation of data for the work; drafting the work or revising it critically for important intellectual content; final approval of the version to be published
YV	Substantial contributions to the conception or design of the work; or the acquisition, analysis, or interpretation of data for the work; final approval of the version to be published
HY	Substantial contributions to the conception or design of the work; or the acquisition, analysis, or interpretation of data for the work; drafting the work or revising it critically for important intellectual content; final approval of the version to be published
MC	Substantial contributions to the conception or design of the work; or the acquisition, analysis, or interpretation of data for the work; final approval of the version to be published
AY	Substantial contributions to the conception or design of the work; or the acquisition, analysis, or interpretation of data for the work; drafting the work or revising it critically for important intellectual content; final approval of the version to be published
IK	Drafting the work or revising it critically for important intellectual content; final approval of the version to be published
IY	Drafting the work or revising it critically for important intellectual content; final approval of the version to be published
AE	Substantial contributions to the conception or design of the work; or the acquisition, analysis, or interpretation of data for the work; final approval of the version to be published
DC	Drafting the work or revising it critically for important intellectual content; final approval of the version to be published
KE	Drafting the work or revising it critically for important intellectual content; final approval of the version to be published
AD	Drafting the work or revising it critically for important intellectual content; final approval of the version to be published
